# Poly[aqua­(dimethyl sulfoxide)(μ_4_-pyridine-2,5-dicarboxyl­ato)calcium(II)]

**DOI:** 10.1107/S1600536810054334

**Published:** 2011-01-15

**Authors:** Hoda Pasdar, Zahra Safari, Hossein Aghabozorg, Behrouz Notash, Masoud Mirzaei

**Affiliations:** a Department of Chemistry, Islamic Azad University, North Tehran Branch, Tehran, Iran; b Department of Chemistry, Shahid Beheshti University, G. C., Evin, Tehran, 1983963113, Iran; c Department of Chemistry, School of Sciences, Ferdowsi University of Mashhad, Mashhad, Iran

## Abstract

In the polymeric title compound, [Ca(C_7_H_3_NO_4_)(H_2_O)(C_2_H_6_OS)]_*n*_, the Ca^II^ ion is coordinated in a distorted penta­gonal–bipyramidal CdNO_6_ geometry. The crystal packing is stabilized by O—H⋯O hydrogen bonds and π–π stacking inter­actions between the aromatic rings of pyridine-2,5-dicarb­oxy­late with centroid–centroid distances of 3.6166 (13) Å.

## Related literature

For related coordination polymers involving pyridine-2,5-dicarb­oxy­lic acid, see: Aghabozorg, Derikvand *et al.* (2008 ▶); Aghabozorg, Manteghi & Sheshmani (2008 ▶); Xu *et al.* (2008 ▶); Sun *et al.* (2006 ▶); Çolak *et al.* (2010 ▶); Wang *et al.* (2009 ▶); Xie *et al.* (2009 ▶).
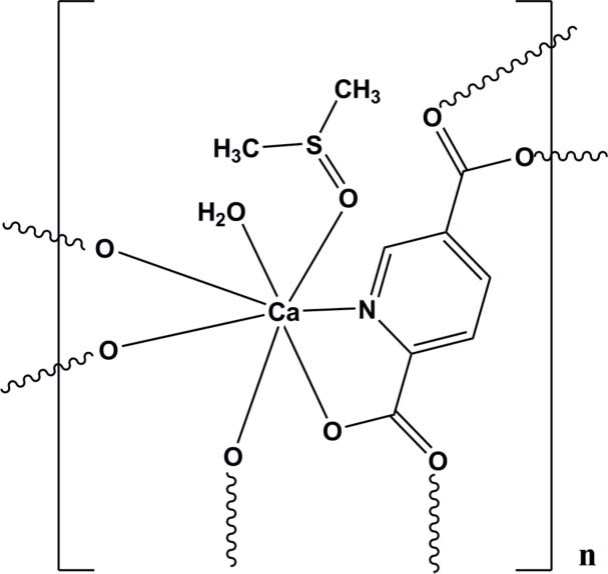


## Experimental

### 

#### Crystal data

[Ca(C_7_H_3_NO_4_)(H_2_O)(C_2_H_6_OS)]
*M* *_r_* = 301.34
Monoclinic, 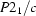

*a* = 10.449 (2) Å
*b* = 11.450 (2) Å
*c* = 10.325 (2) Å
β = 95.93 (3)°
*V* = 1228.7 (4) Å^3^
*Z* = 4
Mo *K*α radiation
μ = 0.70 mm^−1^
*T* = 298 K
0.27 × 0.15 × 0.15 mm

#### Data collection

Stoe IPDS II diffractometer
8616 measured reflections
3302 independent reflections
2718 reflections with *I* > 2σ(*I*)
*R*_int_ = 0.041

#### Refinement

*R*[*F* ^2^ > 2σ(*F* ^2^)] = 0.044
*wR*(*F* ^2^) = 0.097
*S* = 1.11
3302 reflections
173 parameters
H atoms treated by a mixture of independent and constrained refinement
Δρ_max_ = 0.44 e Å^−3^
Δρ_min_ = −0.30 e Å^−3^

### 

Data collection: *X-AREA* (Stoe & Cie, 2005 ▶); cell refinement: *X-AREA*; data reduction: *X-AREA*; program(s) used to solve structure: *SHELXS97* (Sheldrick, 2008 ▶); program(s) used to refine structure: *SHELXL97* (Sheldrick, 2008 ▶); molecular graphics: *ORTEP-3 for Windows* (Farrugia, 1997 ▶); software used to prepare material for publication: *WinGX* (Farrugia, 1999 ▶).

## Supplementary Material

Crystal structure: contains datablocks I, global. DOI: 10.1107/S1600536810054334/bt5441sup1.cif

Structure factors: contains datablocks I. DOI: 10.1107/S1600536810054334/bt5441Isup2.hkl

Additional supplementary materials:  crystallographic information; 3D view; checkCIF report

## Figures and Tables

**Table 1:** Hydrogen-bond geometry (Å, °)

*D*—H⋯*A*	*D*—H	H⋯*A*	*D*⋯*A*	*D*—H⋯*A*
O6—H6*B*⋯O3^i^	0.84 (3)	2.00 (3)	2.782 (2)	155 (3)
O6—H6*A*⋯O1^ii^	0.82 (4)	1.96 (4)	2.739 (2)	158 (3)

Symmetry codes: (i) 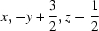; (ii) 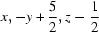.

**Fig. 1.**
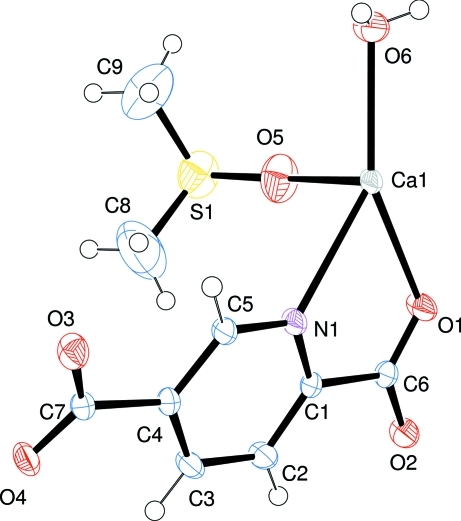
The asymetric unit of title compound with displacement ellipsoids drawn at 50% probability level.

**Fig. 2.**
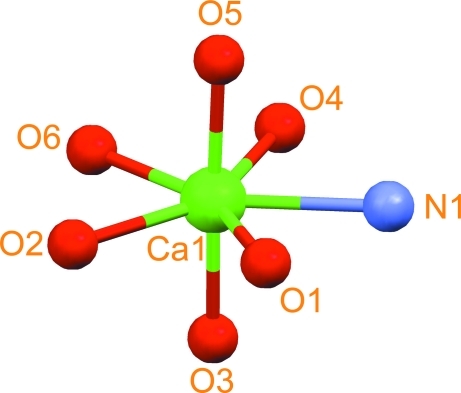
The coordination environment around the Ca(II) ion in the title compound.

**Fig. 3.**
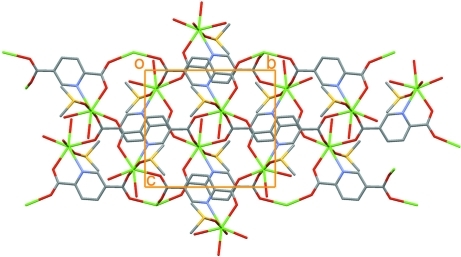
A view of the two-dimensional structure of the title compound down the a-axis. Hydrogen atoms have been ommited for clarity.

**Fig. 4.**
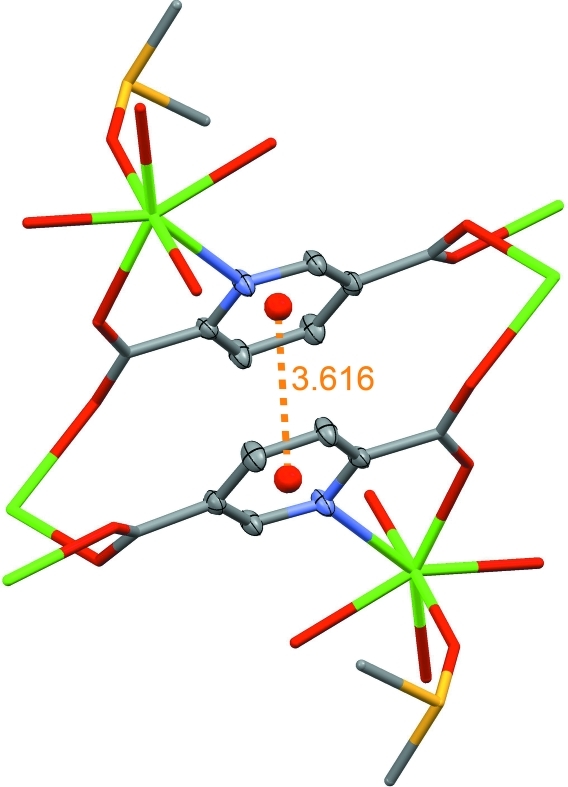
The packing diagram of title compound showing intermolecular π-π interaction (dashed lines) between pyridine rings of py-2,5-dc.

## supplementary crystallographic information

### Comment

Extended frameworks of coordination polymers based on transition metal ions and multifunctional bridging ligands are currently of great interest because of their intriguing topologies and their potential applications (Sun *et al.*, 2006; Wang *et al.*, 2009; Xie, *et al.*, 2009). Pyridine-2,5-dicarboxylic acid (py-2,5- dcH_2_) has unique features because of the presence of two carboxylate groups (O donor atoms) and the pyridine ring (N donor atom), which aids to increase the dimensionality of the assembled covalent network. Therefore, it is most likely that py-2,5-dcH_2_ will form low symmetric structures with metals (Aghabozorg, Derikvand, *et al.*, 2008; Xu *et al.*, 2008; Çolak *et al.*, 2010). Our research group has recently focused on one-pot synthesis of water soluble self-assembly systems that can function as suitable ligands in the synthesis of metal complexes (Aghabozorg, Manteghi & Sheshmani, 2008).

The title compound consists of one deprotonated 2,5-pydc unit, one water molecule, and one dimethylsulfoxide molecule. The asymmetric unit of the title compound is shown in Fig. 1. In the the title compound, Ca^II^ ion is 7-coordinated in a NO_6_ environment. Its geometry is distorted pentagonal bipyramidal. Pentagonal plane is constructed by one oxygen from water, one nitrogen and three oxygen atoms from the (py-2,5-dc)^2–^ and axial positions occupied by two oxygen atoms from (py-2,5-dc)^2–^ and dimethylsulfoxide moieties. A perspective view of the coordination environment around the Ca^II^ ion is shown in Fig. 2.   The crystal structure of title compound shows that the compound is a two-dimensional polymer [Ca(C_7_H_3_NO_4_)(H_2_O)(C_2_H_6_SO)]_n_. The polymeric structure of title compound is shown in Fig. 3. There are O—H···O hydrogen bonds between hydrogen atoms of water molecules and oxygen atoms of py-2,5-dc (Table 2). There is also π–π stacking interactions (Fig. 4) between two aromatic rings of (py-2,5-dc)^2–^ with centroid–centroid distances of 3.6166 (13) Å.

### Experimental

A mixture of CaCl_2_ (0.627 g), pyridine-2,5-dicarboxylic acid (0.1519 g), 1,4-butanediammine (1 ml) in 6 ml DMSO was stirred at room temprature for 2 hrs.The solution was filtered, and the filtrate was stand at room temprature. After two days, colorless block shape crystals of the title compound were obtained (m.p 249°C).

### Refinement

The hydrogen atoms of the water molecule were found in a difference Fourier map and refined isotropically. The C—H protons were positioned geometrically and refined as riding atoms with C—H = 0.93 Å and *U*iso(H) = 1.2 *U*eq(C) for aromatic C—H and C—H = 0.96 Å and *U*iso(H) = 1.5 *Ueq*(C) for methyl groups.

### Crystal data

**Table:**

[Ca(C_7_H_3_NO_4_)(C_2_H_6_OS)(H_2_O)]	*F*(000) = 624.0
*M**_r_* = 301.34	*D*_x_ = 1.629 Mg m^−^^3^
Monoclinic, *P*2_1_/*c*	Mo *K*α radiation, λ = 0.71073 Å
Hall symbol: -P 2ybc	Cell parameters from 3302 reflections
*a* = 10.449 (2) Å	θ = 2.7–29.1°
*b* = 11.450 (2) Å	µ = 0.70 mm^−^^1^
*c* = 10.325 (2) Å	*T* = 298 K
β = 95.93 (3)°	Block, colorless
*V* = 1228.7 (4) Å^3^	0.27 × 0.15 × 0.15 mm
*Z* = 4	

### Data collection

**Table:**

Stoe IPDS II diffractometer	2718 reflections with *I* > 2σ(*I*)
Radiation source: fine-focus sealed tube	*R*_int_ = 0.041
graphite	θ_max_ = 29.1°, θ_min_ = 2.7°
Detector resolution: 0.15 mm pixels mm^-1^	*h* = −14→13
rotation method scans	*k* = −13→15
8616 measured reflections	*l* = −14→14
3302 independent reflections	

### Refinement

**Table:**

Refinement on *F*^2^	Primary atom site location: structure-invariant direct methods
Least-squares matrix: full	Secondary atom site location: difference Fourier map
*R*[*F*^2^ > 2σ(*F*^2^)] = 0.044	Hydrogen site location: inferred from neighbouring sites
*wR*(*F*^2^) = 0.097	H atoms treated by a mixture of independent and constrained refinement
*S* = 1.11	*w* = 1/[σ^2^(*F*_o_^2^) + (0.0423*P*)^2^ + 0.5537*P*] where *P* = (*F*_o_^2^ + 2*F*_c_^2^)/3
3302 reflections	(Δ/σ)_max_ = 0.001
173 parameters	Δρ_max_ = 0.44 e Å^−^^3^
0 restraints	Δρ_min_ = −0.30 e Å^−^^3^

### Special details

**Table:**

Geometry. All e.s.d.'s (except the e.s.d. in the dihedral angle between two l.s. planes) are estimated using the full covariance matrix. The cell e.s.d.'s are taken into account individually in the estimation of e.s.d.'s in distances, angles and torsion angles; correlations between e.s.d.'s in cell parameters are only used when they are defined by crystal symmetry. An approximate (isotropic) treatment of cell e.s.d.'s is used for estimating e.s.d.'s involving l.s. planes.
Refinement. Refinement of *F*^2^ against ALL reflections. The weighted *R*-factor *wR* and goodness of fit *S* are based on *F*^2^, conventional *R*-factors *R* are based on *F*, with *F* set to zero for negative *F*^2^. The threshold expression of *F*^2^ > σ(*F*^2^) is used only for calculating *R*-factors(gt) *etc*. and is not relevant to the choice of reflections for refinement. *R*-factors based on *F*^2^ are statistically about twice as large as those based on *F*, and *R*- factors based on ALL data will be even larger.

### Fractional atomic coordinates and isotropic or equivalent isotropic displacement parameters (Å^2^)

**Table:**

	*x*	*y*	*z*	*U*_iso_*/*U*_eq_	
Ca1	0.67926 (4)	1.08747 (3)	0.15351 (3)	0.01937 (10)	
S1	1.00299 (7)	0.95463 (8)	0.23803 (8)	0.0533 (2)	
O5	0.89654 (19)	1.04175 (19)	0.2093 (2)	0.0525 (5)	
C9	0.9931 (4)	0.8533 (4)	0.1082 (4)	0.0836 (13)	
H9A	0.9068	0.8237	0.0933	0.125*	
H9B	1.0515	0.7899	0.1298	0.125*	
H9C	1.0155	0.8914	0.0308	0.125*	
C8	0.9511 (5)	0.8595 (5)	0.3569 (5)	0.114 (2)	
H8A	0.9386	0.9031	0.4340	0.171*	
H8B	1.0151	0.8003	0.3777	0.171*	
H8C	0.8715	0.8234	0.3237	0.171*	
N1	0.63254 (17)	0.97721 (13)	0.36682 (15)	0.0212 (3)	
C5	0.6057 (2)	0.86315 (16)	0.37822 (17)	0.0207 (4)	
H5	0.5735	0.8228	0.3037	0.025*	
C1	0.67172 (18)	1.03533 (15)	0.47695 (17)	0.0176 (3)	
C3	0.6660 (2)	0.86337 (17)	0.60687 (18)	0.0245 (4)	
H3	0.6803	0.8252	0.6866	0.029*	
C2	0.6872 (2)	0.98248 (17)	0.59788 (18)	0.0237 (4)	
H2	0.7114	1.0262	0.6723	0.028*	
C4	0.62317 (19)	0.80212 (15)	0.49489 (17)	0.0191 (3)	
C7	0.59970 (19)	0.67151 (16)	0.49754 (18)	0.0203 (4)	
O3	0.53823 (15)	0.62711 (12)	0.39822 (14)	0.0265 (3)	
O4	0.64566 (17)	0.61761 (13)	0.59638 (14)	0.0311 (4)	
O2	0.72123 (17)	1.22148 (12)	0.56666 (14)	0.0307 (3)	
O1	0.69341 (17)	1.20480 (12)	0.34958 (13)	0.0307 (3)	
C6	0.6974 (2)	1.16520 (15)	0.46351 (18)	0.0209 (4)	
O6	0.7022 (2)	1.06481 (15)	−0.07874 (15)	0.0350 (4)	
H6A	0.685 (3)	1.127 (3)	−0.116 (3)	0.055 (9)*	
H6B	0.643 (3)	1.019 (3)	−0.106 (3)	0.043 (8)*	

### Atomic displacement parameters (Å^2^)

**Table:**

	*U*^11^	*U*^22^	*U*^33^	*U*^12^	*U*^13^	*U*^23^
Ca1	0.0298 (2)	0.01175 (16)	0.01607 (16)	0.00106 (14)	−0.00007 (13)	0.00002 (13)
S1	0.0306 (3)	0.0622 (5)	0.0649 (5)	0.0089 (3)	−0.0054 (3)	−0.0029 (4)
O5	0.0371 (10)	0.0517 (12)	0.0662 (13)	0.0085 (9)	−0.0062 (9)	−0.0013 (10)
C9	0.077 (3)	0.074 (3)	0.103 (3)	0.005 (2)	0.025 (2)	−0.026 (2)
C8	0.109 (4)	0.128 (4)	0.109 (4)	0.062 (3)	0.038 (3)	0.066 (3)
N1	0.0341 (9)	0.0119 (7)	0.0173 (7)	−0.0012 (6)	0.0011 (6)	0.0000 (5)
C5	0.0321 (10)	0.0128 (8)	0.0169 (8)	−0.0009 (7)	0.0012 (7)	−0.0013 (6)
C1	0.0235 (9)	0.0118 (8)	0.0177 (8)	0.0012 (6)	0.0026 (7)	−0.0012 (6)
C3	0.0383 (11)	0.0187 (9)	0.0159 (8)	−0.0018 (8)	0.0000 (8)	0.0034 (7)
C2	0.0371 (11)	0.0170 (8)	0.0165 (8)	−0.0043 (8)	0.0000 (7)	−0.0022 (7)
C4	0.0252 (9)	0.0122 (8)	0.0200 (8)	0.0005 (7)	0.0034 (7)	0.0013 (6)
C7	0.0280 (10)	0.0133 (8)	0.0200 (8)	0.0001 (7)	0.0049 (7)	0.0014 (6)
O3	0.0342 (8)	0.0172 (6)	0.0272 (7)	−0.0041 (6)	−0.0019 (6)	−0.0016 (5)
O4	0.0573 (10)	0.0150 (6)	0.0203 (7)	0.0015 (6)	−0.0002 (7)	0.0039 (5)
O2	0.0549 (10)	0.0151 (6)	0.0212 (7)	−0.0016 (6)	0.0003 (6)	−0.0042 (5)
O1	0.0598 (10)	0.0131 (6)	0.0191 (6)	−0.0043 (6)	0.0029 (7)	0.0003 (5)
C6	0.0307 (10)	0.0116 (8)	0.0206 (8)	0.0003 (7)	0.0031 (7)	−0.0021 (6)
O6	0.0622 (12)	0.0181 (7)	0.0253 (7)	−0.0083 (8)	0.0076 (7)	−0.0010 (6)

### Geometric parameters (Å, °)

**Table:**

Ca1—O3^i^	2.3249 (16)	C5—C4	1.388 (2)
Ca1—O5	2.343 (2)	C5—H5	0.9300
Ca1—O1	2.4214 (15)	C1—C2	1.382 (3)
Ca1—O2^ii^	2.4215 (15)	C1—C6	1.520 (2)
Ca1—O4^iii^	2.4377 (15)	C3—C2	1.386 (3)
Ca1—O6	2.4484 (17)	C3—C4	1.386 (3)
Ca1—N1	2.6278 (16)	C3—H3	0.9300
Ca1—H6B	2.78 (3)	C2—H2	0.9300
S1—O5	1.501 (2)	C4—C7	1.516 (2)
S1—C8	1.768 (5)	C7—O4	1.246 (2)
S1—C9	1.768 (4)	C7—O3	1.260 (2)
C9—H9A	0.9600	O3—Ca1^iv^	2.3249 (16)
C9—H9B	0.9600	O4—Ca1^v^	2.4377 (15)
C9—H9C	0.9600	O2—C6	1.248 (2)
C8—H8A	0.9600	O2—Ca1^vi^	2.4215 (15)
C8—H8B	0.9600	O1—C6	1.257 (2)
C8—H8C	0.9600	O6—H6A	0.82 (4)
N1—C5	1.344 (2)	O6—H6B	0.84 (3)
N1—C1	1.344 (2)		
			
O3^i^—Ca1—O5	178.05 (7)	S1—C8—H8A	109.5
O3^i^—Ca1—O1	93.30 (6)	S1—C8—H8B	109.5
O5—Ca1—O1	86.82 (7)	H8A—C8—H8B	109.5
O3^i^—Ca1—O2^ii^	87.06 (6)	S1—C8—H8C	109.5
O5—Ca1—O2^ii^	94.87 (7)	H8A—C8—H8C	109.5
O1—Ca1—O2^ii^	79.09 (5)	H8B—C8—H8C	109.5
O3^i^—Ca1—O4^iii^	91.12 (6)	C5—N1—C1	117.08 (16)
O5—Ca1—O4^iii^	87.47 (7)	C5—N1—Ca1	126.80 (12)
O1—Ca1—O4^iii^	137.02 (5)	C1—N1—Ca1	113.85 (11)
O2^ii^—Ca1—O4^iii^	143.87 (5)	N1—C5—C4	123.73 (17)
O3^i^—Ca1—O6	89.31 (7)	N1—C5—H5	118.1
O5—Ca1—O6	91.53 (8)	C4—C5—H5	118.1
O1—Ca1—O6	150.93 (5)	N1—C1—C2	122.99 (16)
O2^ii^—Ca1—O6	72.13 (5)	N1—C1—C6	116.64 (16)
O4^iii^—Ca1—O6	71.77 (5)	C2—C1—C6	120.37 (16)
O3^i^—Ca1—N1	91.41 (6)	C2—C3—C4	118.80 (17)
O5—Ca1—N1	86.89 (7)	C2—C3—H3	120.6
O1—Ca1—N1	64.30 (5)	C4—C3—H3	120.6
O2^ii^—Ca1—N1	143.22 (5)	C1—C2—C3	119.12 (18)
O4^iii^—Ca1—N1	72.87 (5)	C1—C2—H2	120.4
O6—Ca1—N1	144.64 (5)	C3—C2—H2	120.4
O3^i^—Ca1—H6B	78.5 (6)	C3—C4—C5	118.12 (17)
O5—Ca1—H6B	102.0 (6)	C3—C4—C7	121.52 (16)
O1—Ca1—H6B	162.4 (7)	C5—C4—C7	120.34 (17)
O2^ii^—Ca1—H6B	84.9 (7)	O4—C7—O3	126.02 (18)
O4^iii^—Ca1—H6B	59.5 (7)	O4—C7—C4	117.00 (18)
O6—Ca1—H6B	17.1 (7)	O3—C7—C4	116.95 (17)
N1—Ca1—H6B	130.7 (7)	C7—O3—Ca1^iv^	132.07 (13)
O5—S1—C8	105.83 (18)	C7—O4—Ca1^v^	135.17 (13)
O5—S1—C9	107.57 (19)	C6—O2—Ca1^vi^	139.02 (14)
C8—S1—C9	97.1 (3)	C6—O1—Ca1	125.13 (12)
S1—O5—Ca1	151.24 (14)	O2—C6—O1	126.64 (17)
S1—C9—H9A	109.5	O2—C6—C1	116.68 (16)
S1—C9—H9B	109.5	O1—C6—C1	116.68 (16)
H9A—C9—H9B	109.5	Ca1—O6—H6A	110 (2)
S1—C9—H9C	109.5	Ca1—O6—H6B	105 (2)
H9A—C9—H9C	109.5	H6A—O6—H6B	106 (3)
H9B—C9—H9C	109.5		
			
C8—S1—O5—Ca1	−51.3 (4)	C4—C3—C2—C1	−3.5 (3)
C9—S1—O5—Ca1	51.7 (3)	C2—C3—C4—C5	1.1 (3)
O1—Ca1—O5—S1	124.5 (3)	C2—C3—C4—C7	179.23 (19)
O2^ii^—Ca1—O5—S1	−156.7 (3)	N1—C5—C4—C3	2.6 (3)
O4^iii^—Ca1—O5—S1	−12.9 (3)	N1—C5—C4—C7	−175.50 (18)
O6—Ca1—O5—S1	−84.5 (3)	C3—C4—C7—O4	−14.9 (3)
N1—Ca1—O5—S1	60.1 (3)	C5—C4—C7—O4	163.15 (18)
O3^i^—Ca1—N1—C5	89.08 (17)	C3—C4—C7—O3	166.78 (19)
O5—Ca1—N1—C5	−89.97 (18)	C5—C4—C7—O3	−15.2 (3)
O1—Ca1—N1—C5	−177.93 (18)	O4—C7—O3—Ca1^iv^	88.3 (2)
O2^ii^—Ca1—N1—C5	176.05 (15)	C4—C7—O3—Ca1^iv^	−93.54 (19)
O4^iii^—Ca1—N1—C5	−1.66 (16)	O3—C7—O4—Ca1^v^	10.8 (3)
O6—Ca1—N1—C5	−1.7 (2)	C4—C7—O4—Ca1^v^	−167.38 (13)
O3^i^—Ca1—N1—C1	−108.68 (14)	O3^i^—Ca1—O1—C6	103.41 (18)
O5—Ca1—N1—C1	72.27 (14)	O5—Ca1—O1—C6	−74.64 (18)
O1—Ca1—N1—C1	−15.69 (13)	O2^ii^—Ca1—O1—C6	−170.23 (18)
O2^ii^—Ca1—N1—C1	−21.71 (19)	O4^iii^—Ca1—O1—C6	8.2 (2)
O4^iii^—Ca1—N1—C1	160.58 (14)	O6—Ca1—O1—C6	−162.06 (16)
O6—Ca1—N1—C1	160.53 (13)	N1—Ca1—O1—C6	13.44 (16)
C1—N1—C5—C4	−3.8 (3)	Ca1^vi^—O2—C6—O1	37.1 (4)
Ca1—N1—C5—C4	157.96 (15)	Ca1^vi^—O2—C6—C1	−143.44 (16)
C5—N1—C1—C2	1.2 (3)	Ca1—O1—C6—O2	170.04 (17)
Ca1—N1—C1—C2	−162.86 (15)	Ca1—O1—C6—C1	−9.4 (3)
C5—N1—C1—C6	−177.95 (17)	N1—C1—C6—O2	173.06 (18)
Ca1—N1—C1—C6	18.0 (2)	C2—C1—C6—O2	−6.1 (3)
N1—C1—C2—C3	2.4 (3)	N1—C1—C6—O1	−7.5 (3)
C6—C1—C2—C3	−178.49 (18)	C2—C1—C6—O1	173.34 (19)

Symmetry codes: (i) −*x*+1, *y*+1/2, −*z*+1/2; (ii) *x*, −*y*+5/2, *z*−1/2; (iii) *x*, −*y*+3/2, *z*−1/2; (iv) −*x*+1, *y*−1/2, −*z*+1/2; (v) *x*, −*y*+3/2, *z*+1/2; (vi) *x*, −*y*+5/2, *z*+1/2.

### Hydrogen-bond geometry (Å, °)

**Table:**

*D*—H···*A*	*D*—H	H···*A*	*D*···*A*	*D*—H···*A*
O6—H6B···O3^iii^	0.84 (3)	2.00 (3)	2.782 (2)	155 (3)
O6—H6A···O1^ii^	0.82 (4)	1.96 (4)	2.739 (2)	158 (3)

Symmetry codes: (iii) *x*, −*y*+3/2, *z*−1/2; (ii) *x*, −*y*+5/2, *z*−1/2.
